# Delayed payment schemes in Central-Eastern Europe and Middle-East

**DOI:** 10.3389/fmed.2022.940371

**Published:** 2022-08-12

**Authors:** Ildikó Ádám, Marcelien Callenbach, Bertalan Németh, Rick A. Vreman, Johan Pontén, Tea Strbad, Dalia Dawoud, Alexander Kostyuk, Ahmed Seyam, László Nagy, Wim G. Goettsch, Zoltán Kaló

**Affiliations:** ^1^Center for Health Technology Assessment, Semmelweis University, Budapest, Hungary; ^2^Division of Pharmacoepidemiology and Clinical Pharmacology, Utrecht Institute for Pharmaceutical Sciences (UIPS), Utrecht University, Utrecht, Netherlands; ^3^Syreon Research Institute, Budapest, Hungary; ^4^National Health Care Institute Zorginstituut Nederland (ZIN), Diemen, Netherlands; ^5^The Dental and Pharmaceutical Benefits Agency Tandvårds-Läkemedelförmånsverket (TLV), Stockholm, Sweden; ^6^Croatian Health Insurance Fund, Zagreb, Croatia; ^7^National Institute for Health and Care Excellence (NICE), London, United Kingdom; ^8^National Research Center for Health Development, Ministry of Health (MoH), Nur-Sultan, Kazakhstan; ^9^Universal Health Insurance Authority, Cairo, Egypt

**Keywords:** managed entry agreements (MEA), reimbursement, delayed payment, value-based pricing, pay for performance, outcome-based payment, spread payments

## Abstract

The need for innovative payment models for health technologies with high upfront costs has emerged due to affordability concerns across the world. Early technology adopter countries have been experimenting with delayed payment schemes. Our objective included listing potential barriers for implementing delayed payment models and recommendations on how to address these barriers in lower income countries of Central and Eastern Europe (CEE) and the Middle East (ME). We conducted a survey, an exploratory literature review and an iterative brainstorming about potential barriers and solutions to implement delayed payment models in these two regions. A draft list of recommendations was validated in a virtual workshop with payer experts from the two regions. Eight barriers were identified in 4 areas, including transaction costs and administrative burden, payment schedule, information technology and data infrastructure, and governance. Fifteen practical recommendations were prepared to address these barriers, including recommendations that are specific to lower income countries, and recommendations that can be applied more universally, but are more crucial in countries with severe budget constraints. Conclusions of this policy research can be considered as an initial step in a multistakeholder dialogue about implementing delayed payment schemes in CEE and ME countries.

## Introduction

The focus of research and development in health care has been changing recently. As opposed to bringing new technologies to the marketplace in large disease groups typically managed in primary care, innovators are focusing more on smaller target patient groups in specialty diseases (e.g., oncology, hematology, autoimmune diseases) or rare diseases. The complexity of new technologies has also increased, initially with the uptake of biological medicines, followed by combined personalized solutions (e.g., molecular diagnostics and precision medicines or pharmaceuticals supported by digital health solutions) and most recently with cell and gene therapies.

Previous research summarized innovative payment models for new health technologies, including those that might be able to manage the market access of potentially curative health technologies. These technologies may have the potential to be cost-effective, as they might prevent chronic treatments and negative clinical outcomes in the long-run. However, due to the high upfront costs health care payers need to find a solution for two different problems, (1) how to manage the uncertainty around whether long-term effects will be realized, and (2) how to overcome challenges of managing the short-term budget impact of these therapies (1).

**Figure 1:**
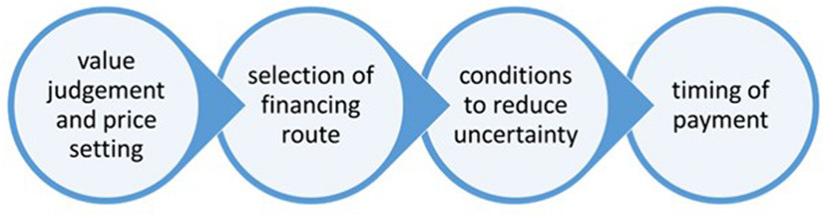
Components of complex payment models.

As health care payers need to address multiple challenges, they may apply complex payment models, as described in Figure 1. The first component of complex payment models may include extended evaluation frameworks, which covers patient centric and societal value criteria in addition to traditional value judgement based on incremental health gain and health care costs. The second component may be consideration of a special financing route as opposed to the positive reimbursement list, such as joint international procurement, financing only in hospitals, or reimbursement on a named-patient basis. The third component of innovative payment models may describe special conditions for public financing of new technologies, such as financial or outcome based risk-sharing agreements, or restricted prescription only in specialty health care centers. The fourth component may improve the sustainability of health care financing by introducing delayed payment models.

The importance of this fourth component has been highlighted with the introduction of human papilloma virus vaccinations, direct antiviral agents to treat hepatitis C or Advanced Therapy Medicinal Products (2). These new health therapies are single interventions with significant short-term budget impact, which may be compensated by long-term health gain and avoided future health care costs (3). Due to the high initial costs the timing of payment is a critical question for health care payers to manage their budget. Instead of the standard upfront payment methods, in which manufacturers receive the payment from health care payers at the time of delivering the treatment, different types of delayed payment options were described by Vreman et al. (4). These include (a) paying treatment costs only after results have been achieved, or (b) annuity or staggered payment methods, in which payments are spread over multiple years with an agreement upon amount of treatment or outcomes delivered, and (c) health leasing or subscription methods, in which payment is made for the unlimited use of a therapy within a predefined period. All these delayed payment options can be implemented at patient or population level.

While a wide range of managed entry agreements have been extensively used in Central and Eastern Europe (CEE) and the Middle-East (ME) (5, 6), outcome based risk-sharing agreement to manage the uncertain effectiveness or safety (i.e., the third component on Figure 1), or delayed payment methods to manage the affordability (i.e., the fourth component on Figure 1) of new health technologies with high upfront costs were mainly described in developed countries.

As part of the European Commission funded HTx H2020 project, our objective was to explore the transferability of both outcome-based and delayed payment methods for technologies with high upfront costs to lower income developing countries within and outside the European Union with special focus on countries in CEE and ME. The transferability assessment included listing barriers for implementing outcome-based reimbursement and making recommendations on how to address these barriers have been described in a previous paper (7), while this paper summarizes the barriers and potential solutions for implementing delayed payment models focusing on the perspective of public health care payers. On the other hand, if health care payers can improve the patient access to health technologies with high upfront cost by implementing delayed payment models, it eventually has positive impact on all different stakeholders, including patients, health care providers and manufacturers.

## Methods

The methodology for listing barriers and making recommendations for implementing delayed payment methods in Central and Eastern Europe or in the Middle East was the same as for outcome-based reimbursement, and so it can be read in detail in a separate manuscript (7).

In short, once the description of different types of delayed payment models was completed by Vreman et al. (4), the process continued with exploring the potential barriers and drafting recommendations for innovative payment models by (1) conducting a survey in CEE and ME countries, (2) reviewing the scientific and gray literature, and (3) holding iterative discussions within HTx consortium members. The survey covered four topics on reimbursement and payment models in different countries, and one topic was dedicated to the current and future use of delayed payment models (see Appendix 1). The approach and methodology of the survey along with the results are summarized in a separate manuscript (8).

As a next step a virtual workshop was organized to review the draft list of barriers and recommendations in June 2021 with 16 members of the HTx consortium and 14 payer experts from 15 countries in Central and Eastern Europe and Middle East. Finally, the draft report with consolidated list of barriers and recommendations was circulated among workshop participants, who made final comments and amendments to the report.

While the methodology for listing barriers and making recommendations for outcome-based reimbursement and delayed payment models was the same, the process, especially the literature review and iterative discussions within the research team, was kept separate.

## Results

After deduplication of barriers retrieved from different sources, and the final list of 8 different barriers were presented in four groups, including (i) transaction costs and administrative burden, (ii) payment schedule, (iii) IT and data infrastructure, and (iv) governance.

To address all different barriers overall 15 practical recommendations were made by consensus of experts from multiple countries as described in Table 1. The barriers are formulated from the perspective of the public health care payers.

**Table 1:** Summary of barriers and recommendations focusing on the perspective of public health care payers.

**Group of barriers**	**Barriers**	**Summary of recommendations**
Transaction costs and administrative burden	Complex and resource intensive negotiations on contractual terms (including the first agreement and renegotiations)	1) Consider transferring the structure of existing agreements from higher income countries 2) Develop contract archetypes for most common schemes 3) When agreements are renegotiated, the latter agreement should be simpler than the first 4) Re-opener clauses of agreements after entry of competitive product
	Costly implementation of agreements with delayed payment	1) Rely on existing infrastructure 2) Reuse of existing claims or medical data 3) In the long-run, adjust payer's data infrastructure to such agreements
Payment schedule	Limited experience with determining the optimal amount and/or duration of payments	1) Greater dialogue between payers and HE&OR experts 2) Consider transferring the structure of existing agreements from higher income countries 3) Develop contract archetypes for most common schemes 4) When agreements are renegotiated, the latter agreement should be simpler than the first 5) Consider that upfront payment has higher present value than delayed payment
	Conflicting financial flows for both parties (i.e., public health care payers and manufacturers) due to 12-month budgetary cycles	Propose changes to European and national accounting rules (e.g., to allow accruals over several years)
IT and data infrastructure	Failure to monitor the patient status with current infrastructure	1) If difficulties to collect data is expected, consider a pilot phase with adjustment according to early experiences 2) In the long-run adjust data infrastructure of health care payers to such agreements
	Limited uptake of patient registries	Facilitate the establishment of patient registries
Governance	Lack of regulation	1) Review regulatory frameworks in higher income countries 2) Consider the implementation of pilot cases, and prepare regulatory legal framework based on experiences in the pilot phase
	Weakness of public sector to efficiently negotiate with multinational industry	1) Consider transferring the structure of existing agreements from higher income countries 2) Strengthen HTA system to promote value for money and affordability concepts 3) Joint procurement by smaller countries to increase the purchasing power

Some of the barriers were related to limited capacities and constraints of health care payers in CEE and ME countries for implementing complex payment models, so 5 barriers (and related recommendations) of delayed payment models to improve the affordability of technologies with high upfront costs were also listed among barriers of implementing outcome-based reimbursement with general descriptions and specific details. The other 3 barriers were specific to specific delayed payment models.

### Barriers of implementing delayed payment models in CEE and ME countries from the perspective of health care payers

Two challenges were described related to high transaction costs and administrative burden of delayed payment models. Compared with upfront payment models, solutions for delayed payment are associated with complex and resource intensive negotiations on contractual terms, including not only the initial agreement but renegotiation of terms after the first contract is terminated. Secondly, the implementation of these agreements also requires significant resources, as the timing of service provision is not linked to the timing of payments.

Barriers could also be attributable to the payment schedule. Until sufficient experience is accumulated from delayed payment agreements for several different types of health technologies, both payers and manufacturers of health technologies need to take significant risks with determining the optimal amount and duration of payments. Even if there is an agreement on spreading the payment to more periods, it may result in conflicting financial flows with current accounting practices and regulations for both parties. Budget holders mostly have to consider 12-month budgetary cycles, while manufacturers should strictly follow international and national accounting rules and reflect revenues and liabilities annually (9).

Delayed payment schemes are often linked to outcome-based agreements, especially when continuing payments after treatment failure makes no sense. Current information technology (IT) and data infrastructure is prone to failure to monitor the patient status. In fact, collecting, organizing or accessing data are the ones of the most frequently reported barriers of implementing outcome-based agreements by the public health care payers, which is often linked with delayed payment schemes (9). Patient registries may alleviate the burden of data collection, however, in CEE and ME countries the availability and uptake of such registries is limited.

The final group of barriers is related to governance of public health care systems. First, current legal frameworks may not be appropriate to accommodate delayed payment schemes. And if this problem is solved, civil servants in national public sectors may not be prepared and incentivised to efficiently negotiate with multinational industry. This is especially true in countries with relatively small market potential, where headquarters of multinational companies may have limited interest in approving unique local proposals.

### Recommendations for implementing delayed payment models in CEE and ME countries

Several practical recommendations were made to facilitate the adoption of delayed payment models in lower-income CEE and ME countries. Some recommendations may be a solution for multiple barriers, the connections between barriers and recommendations are presented in Table 1.

#### Recommendation #1-consider transferring the structure of existing agreements from higher income countries

Lower income countries (including CEE and ME countries) can benefit from experiences of higher income countries with delayed payment models. While some information may also be in the public domain on potential barriers and related solutions, direct exploratory discussion with health care payers in forerunner countries and manufacturers is also advocated. It has to be noted that transferring solutions from other jurisdictions without adjustment to local environment may not be feasible, especially if there are major differences in health care delivery systems and treatment practices. However, existing structures from elsewhere may be a good starting point in designing the structure of delayed payment schemes.

Similarly, review of practices and solutions of forerunner countries to adjust the regulatory and legislative framework to accommodate delayed payment schemes could be highly beneficial in lower income countries.

#### Recommendation #2-pharmaceutical industry should develop contract archetypes for most common schemes

Multinational manufacturers of health technologies should develop a master document that can describe the adaptability of delayed payment methods to different archetypes of health care systems. Development of common solutions for similar systems can accelerate preparations for offerings and negotiations in different countries, and prevent failures related to “one size fits all” market access strategies. While the master document should be updated on a continuous basis with new experiences, too detailed description of contracts would result in the applicability of such schemes only to individual countries.

#### Recommendation #3-when agreements are renegotiated, the latter agreement should be simpler than the first

As delayed payment agreements have to be renegotiated after their termination, there is an opportunity to simplify the original conditions based on the experiences in the initial period, which can be facilitated by entry and exit criteria. Real world effectiveness data can help to clarify the expected payments in the second or third years after therapy initiation.

#### Recommendation #4-apply re-opener clauses of agreements after entry of competitive product

Recognition of market dynamics should be reflected in agreements with several years of duration. Therefore, it is recommended to add re-opener clauses to the agreement for the market launch of competitive technologies (competitive technologies term should also be defined in the agreement whether it would be based on ATC code, indication, etc.) or, if applicable, for the patent expiry of the health technology. Alternatively, for such cases a pre-defined adjustment of the payment may be considered (4).

#### Recommendation #5–in the short run, rely on existing infrastructure

Implementation of delayed payment schemes can be fairly complex, expensive and unreliable (e.g., due to inclusion bias), if it necessitates the development of a new infrastructure (including data reporting system or data lakes) for its administration. Therefore, it is highly recommended that such schemes should rely on existing infrastructure.

#### Recommendation #6-in the long run, adjust data infrastructure of health care payers to such agreements

Initial failures to monitor the patient status with current infrastructure can be considered as a need for changing the data infrastructure of health care payers. While changing the infrastructure cannot be justified based on a single case, in the long-run more and more potentially curative technologies with high upfront costs can be expected, therefore adjustment of the data infrastructure to accommodate delayed payment options in addition to alignment with international data standards is an inevitable step in the long-run.

#### Recommendation #7-reuse of existing claims or medical data

Reusing existing claims data or electronic medical records for administering delayed payment schemes reduces the human or financial burden of implementation. Linking existing databases–e.g., patient registries with payer's databases–may require additional investment, however, the availability of such joint databases may open further opportunities in generating real world evidence to improve health policies. It should also be noted that reusing existing data for multiple purposes has the potential to increase data quality.

#### Recommendation #8-greater dialogue between payers and HE&OR experts

Health economics and outcomes research (HE&OR) experts, researchers in academic centers within and outside a country may accumulate broad experiences from previous or ongoing research projects (such as the current HTx H2020 project), while HE&OR experts at multinational companies can draw negative and positive conclusions from establishing similar agreements in many different countries. In many CEE and ME countries there is little room for information exchange between payers and HE&OR experts, which may prevent knowledge transfer from research projects and generalization of learnings from previous agreements. Greater dialogue between payers and HE&OR experts may improve the information exchange, contributes to build trust and has the potential to optimize payment schedules.

#### Recommendation #9-consider that upfront payment has higher present value than delayed payment

According to methodological guidelines there is no need to apply discount rates in budget impact analyses (10). As opposed to these standards, timing of payments should be reflected in the agreements of delayed payment models, and so an appropriate discount factor should be applied to calculate the present value of future payments. Possibly a third party may be involved to mitigate financial risks of spread payments. Other studies elaborated more on the alternatives where a financial service is provided by a third party (11).

#### Recommendation #10-propose changes to international and national accounting rules (e.g., to allow accruals over several years)

Pharmaceutical companies and health care payers face challenges in spreading payments over a certain period due to national and European accounting rules. Therefore, a complex approach would be essential that would enable parties to choose spread or delayed payment. Maes et al. concluded that European System of Accounts (“ESA”) is a real barrier in implementing annuity payments. Annuity payments should be recognized as debt in the year of delivering treatment, which has an impact on the government's deficit in the year of treatment (12). Hence, a general proposal to enable the international and national regulations allowing payments division over a certain period would improve the adaptability of delayed payment schemes to the accounting systems of both health care payers and multinational companies.

#### Recommendation #11-if difficulties to collect data is expected, consider a pilot phase with adjustment according to early experiences

In any novel policy solution, it makes sense to introduce a pilot period with strict monitoring process of early experiences. The pilot phase would provide opportunity to adjustment in the first couple of agreements according to early experiences.

#### Recommendation #12-consider the implementation of pilot cases

Similarly to the pilot cases in individual agreements, pilot cases should also be considered before making changes in the regulatory and legislative framework to accommodate delayed payment schemes.

#### Recommendation #13-facilitate the establishment of patient registries

Collection of real-world health outcomes data in patient registries is advocated for many reasons, and implementation of delayed payment schemes can also benefit from the establishment of patient registries. Training of health care professionals, manufacturers and payers is key in overcoming interpretation and analysis bias (12). Finally, enabling multi country cooperation could help in decreasing the burden of setting-up registries and eliminate duplicating the work of collecting data.

#### Recommendation #14-strengthen HTA system to promote value for money and affordability concepts

HTA facilitates policy decisions based on the best available evidence related to multiple criteria. Delayed payment schemes can improve the affordability of health technologies with high upfront costs in parallel with supporting value-based health care. Budget impact analyses may help to quantify how such schemes can contribute to the sustainability of health care financing. A prerequisite for informed decision-making around delayed payment models within a value-based health care environment is a strong HTA system.

#### Recommendation #15-joint procurement by smaller countries to increase the purchasing power

A few years ago, the MEAT (Most Economically Advantageous Tender) value framework concept was introduced, and a discussion started whether it could be a useful tool in purchasing high-cost health technologies jointly by multiple countries. The concept advocates the consideration of those health technologies-instead of the cheapest alternatives-that can bring benefits to the economy on a wider scale, to different stakeholders in the health systems, including patients, providers and health professionals, while taking into account advantageous financial solutions (13). Delayed payment models for potentially curative technologies with high upfront costs can be a relevant subject for the MEAT framework.

## Discussion

This study provided a consensus statement on important barriers related to delayed payment schemes in CEE and ME countries and practical recommendations to overcome those barriers. Some recommendations are specific only to lower income countries, while other recommendations apply more universally, but are especially crucial in developing countries.

The focus on CEE and ME countries is especially important for two reasons. At first, experiences about delayed payment schemes have been published about higher income countries, which may not be fully transferable to developing countries. Second, populations of these countries have poorer health status, so demand for potentially curative health technologies is greater. On the other hand, financial resources are more limited, and improving sustainability of health care financing with delayed payment schemes may result in even more value in these countries.

Similarly to recommendations to implement outcome-based reimbursement to reduce the uncertainty in the effectiveness and safety of new technologies in CEE and ME countries, recommendations described in this paper on implementing delayed payment schemes can be considered as an initial step in a multistakeholder dialogue, and continuation of this work is highly recommended. Since conducting research in a pandemic period reduced the opportunity of organizing face-to-face focus group meetings. Similarly, initiation of discussions with health care payers, who were overwhelmed with managing health care financing in a difficult health and economic period, was challenging.

## Limitations

The most important limitation of our research is that only a limited number of CEE and ME countries were included in the workshop and survey addressing the barriers and recommendations of delayed payment schemes. Although a small group of experts participated at our workshops, they had thorough experience about the health care financing system of their own countries.

In the current research we focused on listing the barriers and recommendations that could be relevant in the lower income countries of the CEE and ME region. The recommendations were formulated from the perspective of health care payers from multiple countries. The overall impact of recommendations and their feasibility on a country level should be explored in future studies.

Although barriers and recommendations were considered relevant across all participant countries, they were not ranked by their importance, because such prioritization need to be country specific. This could be a further research topic in this area.

## Data availability statement

The original contributions presented in the study are included in the article/supplementary material, further inquiries can be directed to the corresponding author.

## Author contributions

All authors listed have made a substantial, direct, and intellectual contribution to the work and approved it for publication.

## Funding

The HTx project has received funding from the European Union's Horizon 2020 research and innovation program under grant agreement N°825162. The content of this paper reflects only the HTx group's views and the European Commission is not liable for any use that may be made of the information contained herein. The article was prepared with the professional support of the Doctoral Student Scholarship Program of the Co-operative Doctoral Program of the Ministry of Innovation and Technology financed from the National Research, Development and Innovation Fund, Hungary under grant agreement N°1012597.

## Conflict of interest

The authors declare that the research was conducted in the absence of any commercial or financial relationships that could be construed as a potential conflict of interest.

## Publisher's note

All claims expressed in this article are solely those of the authors and do not necessarily represent those of their affiliated organizations, or those of the publisher, the editors and the reviewers. Any product that may be evaluated in this article, or claim that may be made by its manufacturer, is not guaranteed or endorsed by the publisher.

## Author disclaimer

The views expressed in this article are the personal views of the authors and may not be understood or quoted as being made on behalf of or reflecting the position of the agencies or organizations with which the authors are affiliated.

## Figures and Tables

**Table:** 

	**Never applied**	**Rarely applied**	**Often applied**	**Always applied / Mandatory to apply**
Upfront payment to the manufacturer				
Payments at outcome achieved				
Annuity payments				
Health leasing / subscription				

**Table:** 

	**Never applied**	**Rarely applied**	**Often applied**	**Always applied / Mandatory to apply**
Upfront payment to the manufacturer				
Payments at outcome achieved				
Annuity payments				
Health leasing / subscription				

**Table:** 

**Payment model**	**Barriers preventing its use (please list 1-3 barriers in each row)**
Payments at outcome achieved	
Annuity payments	
Health leasing / subscription	

## Appendix 1

Extract from the survey regarding use of payment models.

Topic 4. Use of payment models.

1. How often are each of the following payment models currently being applied in your country for inpatient (in-hospital) pharmaceuticals? Please select one option in each row.

2. Is there a difference between inpatient and outpatient pharmaceuticals in the types of payment models that are applied in your country?
a.No
b. Yes, namely ….
c. Other
3. How often are each of the following payment models currently being applied in your country for outpatient (out-hospital) pharmaceuticals? Please select one option in each row.

1. 4. Which of the following payment models would you prefer to be applied in your country more often than currently, 5 years from now? (multiple choices are possible)
  a. Upfront payment to the manufacturer
  b. Payment at outcome achieved
  c. Annuity payment
  d. Health leasing / subscription
  e. Other, namely….
2. Upfront payment may not be a viable option for the most expensive treatments. In your opinion what are the greatest barriers preventing the use of the following potential payment models?
